# Dr. Thomas A. O’Hare

**Published:** 1916-12

**Authors:** 


**Dr. Thomas A. O'Hare,** Pennsylvania 1875, died at his home in Rochester, Nov. 20, in his 68th year. He was born and educated in Rochester and had practiced there all his active life. He had been president of the local Academy of Medicine, Pathological Society, and Monroe Co. Medical Society and of the Medical Assn. of the State of N. Y. He was, at the time of his death, President of the staff of St. Mary's Hospital and of the Board of Managers of the Rochester State Hospital and Physician to St. Patrick's Girls' Orphan Asylum. As thus indicated, he was prominently interested in philanthropy and in medical organizations. As a physician he was universally recognized as conscientious and skillful.

